# Sequential chemotherapy followed by radical thoracic radiotherapy (50 Gy in 25 fractions) in limited stage small cell lung cancer

**DOI:** 10.3332/ecancer.2020.1019

**Published:** 2020-03-09

**Authors:** Muhammad Shahid Iqbal, Joseph Carlow, Fiona McDonald, Philip Atherton, Helen Turnbull, Sandeep Singhal, Timothy Simmons, Paula Mulvenna, Josef Kovarik, Rhona McMenemin, Jill Gardiner, Alastair Greystoke

**Affiliations:** 1 Department of Clinical Oncology, Northern Centre for Cancer Care, The Newcastle upon Tyne Hospitals NHS Foundation Trust, Newcastle upon Tyne, NE7 7DN, United Kingdom; 2 Newcastle University, Newcastle upon Tyne, United Kingdom; 3 Department of Clinical Oncology, Aberdeen Royal Infirmary, Aberdeen, AB25 2ZN, United Kingdom; 4 Department of Medical Oncology, Northern Centre for Cancer Care, The Newcastle upon Tyne Hospitals NHS Foundation Trust, Newcastle upon Tyne, NE7 7DN, United Kingdom

**Keywords:** small cell lung carcinoma, sequential chemotherapy, thoracic radiotherapy

## Abstract

**Introduction:**

For limited stage small cell lung cancer (LS-SCLC) where concurrent chemoradiotherapy is inappropriate due to tumour bulk, co-morbidities or performance status, sequential treatment using chemotherapy followed by radiotherapy is the standard of care. The outcomes are not well established; we assessed in a single institution, the survival of these patients, prognostic factors and compared to the limited existing literature.

**Materials and Method:**

Retrospective data was collected on all 59 patients diagnosed with LS-SCLC from 2011 to 2016 who received chemotherapy consisting of Carboplatin or Cisplatin + Etoposide followed by thoracic radiotherapy (50 Gy in 25 fractions) +/- prophylactic cranial irradiation (PCI).

**Results:**

Median age at diagnosis was 66 years (range 46–90). Patients received a median of four cycles of chemotherapy (range: 1–6) and all the patients completed a full course of radiotherapy with only one patient experiencing grade >2 radiation induced toxicity. With a median follow up of 20.6 months, 45 patients had died with a median survival of 20.6 months. 2-year overall survival (OS) rates were 42%. Age using a cut-off of 65 was prognostic (median OS 25.6 months ≤65 years versus 14.1 months >65 years, p = 0.013) but gender, stage and receipt of PCI were not.

**Conclusions:**

Most of the literature reporting outcome from sequential treatment in LS-SCLC is old and used a variety of radiotherapy regimens. Our data shows inferior outcomes to the gold standard concurrent chemoradiotherapy but support its usage in less fit patients with reasonable outcome observed.

## Introduction

Lung cancer is the third most common cancer amongst males and females in the UK [1] with 39,041 new cases diagnosed in 2016 [2]. Of these 39,041 cases small cell lung cancer makes up 10%–15% [2]. Small cell lung cancer (SCLC) typically presents at an advanced stage with two thirds of patients having distant metastases at the time of presentation [3], while SCLC should be staged according to the TNM (Tumour, Node, Metastases) classification; treatment may often be guided by whether the cancer is considered limited or extensive in stage using the US Veteran Association definition [4] where limited disease can be safely included in a radical radiotherapy field.

For limited stage small cell lung cancer (LS-SCLC) concurrent chemoradiotherapy (cCRT) is the standard of care, however, in patients who are deemed inappropriate for cCRT due to co-morbidities or tumour bulk, sequential therapy using chemotherapy followed by radical thoracic radiotherapy (TRT) can still result in cure and may be considered as a suitable treatment option. The research into LS-SCLC treated with sequential chemoradiotherapy (seqCRT) is sparse with multiple regimens reported.

The aim of this single-centre retrospective study is to analyse the survival of patients treated with sequential chemotherapy (cisplatin or carboplatin and etoposide) followed by TRT 50 Gy in 25 daily fractions, and the factors affecting the survival.

## Materials and methods

Fifty nine consecutive patients, between September 2011 to August 2016 with biopsy proven LS-SCLC, treated with platinum and etoposide followed by TRT 50 Gy in 25 daily fractions were included. Data were collected retrospectively from patient records. All patients had staging investigations including computed tomography (CT) thorax abdomen and brain using TNM 7th edition, histological verification with either bronchoscopy ± bronchial lavage, CT guided biopsy or endobronchial ultrasound, pulmonary lung function test, routine blood tests and ethylenediamine tetraacetic acid based glomerular function rate measurement. World Health Organisation (WHO) performance status (PS) was recorded for all patients.

### Chemotherapy

Chemotherapy regimen consisted of 21 days cycle of combination of carboplatin (area under curve 5) on day 1 or cisplatin 75 mg/m^2^, with etoposide 120 mg/m^2^ intravenous infusion on day 1 followed by etoposide 100 mg twice day orally on days 2 and 3. The aim was to give four to six cycles of chemotherapy depending on tolerability, disease response and clinician’s choice.

### Radiotherapy

TRT was started three to four weeks after last cycle of chemotherapy. All patients underwent a contrast enhanced 3D CT scan for radiotherapy planning, comprising 3 mm slice thickness in supine position. A gross tumour volume (GTV) was drawn on each slice if there was residual/visible disease post chemotherapy. A clinical tumour volume (CTV) comprised a 5-mm margin of GTV radiologically in all the directions. The aim was to cover post chemotherapy lung disease and pre-chemotherapy nodal disease in CTV by manual adjustment. Similar manual adjustment of CTV was permitted when disease adjacent to a structure such as a vertebra was not thought to invade the structure. Planning target volume (PTV) was then obtained using a further 10 mm margin craniocaudally and 8 mm laterally. The radiation dose of 50 Gy was prescribed to the isocentre and was delivered in 25 daily fractions.

For prophylactic cranial irradiation (PCI), thermoplastic immobilisation device was made for immobilisation and the target volume was whole brain. Two regimens, either 25 Gy in 10 daily fractions or 20 Gy in 5 daily fractions were used (depending on clinician’s choice) using two lateral parallel opposed fields.

### Statistics

Overall survival was calculated from the date of diagnosis to either date of death or censoring on the date last seen. Statistical analysis was performed using SPSS (Version 24). Survival was calculated using the Kaplan–Meier method, with assessment of the differences in survival using log rank comparison and Cox regression analysis. The outcomes of interest were overall survival (OS) and progression free survival (PFS). Fisher’s exact test was used to assess the association between use of PCI and the development of brain metastases. A *p* value of <0.05 was considered as statistically significant.

### Literature search

A literature search was performed in Medline and EMBASE using the keywords sequential, late/delayed radiation, chemoradiotherapy, chemoradiation, small cell lung cancer, SCLC, limited stage. References of the studies identified were then searched for further studies to be included. Studies involving exclusive concurrent chemoradiotherapy were excluded.

### Ethics consideration

This retrospective study was registered with the local hospital clinical effectiveness register as a service review project.

## Results

The total number of patients who received treatment was 59. The median age at diagnosis was 66 years (range 46–90). The male to female ratio was 30:29. In 18 patients (30.5%), CT-PET scan (positron emission tomography) was also available for staging. Fifty four patients (91%) were stage III, 4 (7%) were with stage II and 1 patient (2%) was with stage I.

There were multiple reason(s) why patients did not receive CRT which have been grouped into five separate categories as shown in Table 1 with the most common being co-morbidities or poor performance status.

Patients received a median of four cycles of platinum-based doublet chemotherapy, carboplatin or cisplatin/etoposide (range 1–6). Two patients (3.4%) received cisplatin instead of carboplatin. All 59 patients completed a subsequent full course of TRT, 50 Gy in 25 fractions. Radiotherapy was well tolerated with only one patient experienced grade III toxicity of pyrexia of unknown origin, hyponatraemia and confusion. Forty six patients (78%) received PCI. Four patients (7%) declined PCI, age greater than 75 was felt to be a contraindication in two patients and in one patient (2%), PCI was not offered due to declining performance status. In the remaining six patients (10%) who didn’t receive PCI, no reason was recorded.

With a median follow up of 20.6 months, 45 patients (76%) had died and disease had recurred in 33 patients (56%). In 25 cases (76% of the patients with disease recurrence), there were distant metastases and in remaining 8 patients (24%), disease recurred locoregionally only. Of seven patients (21% of the relapsed patients) who developed brain metastases; four patients had received PCI whilst three had not received PCI (*p* = 0.093). Twenty seven patients received further treatment, which included re-challenge platinum etoposide chemotherapy or oral topotecan therapy. None received radical re-irradiation.

**Figure 1.**
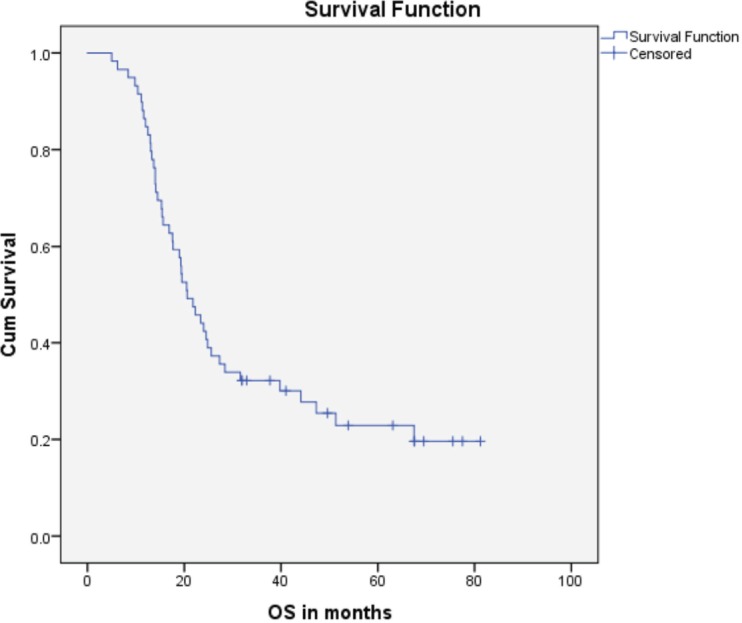
Kaplan–Meier curve for overall survival (in months).

**Figure 2.**
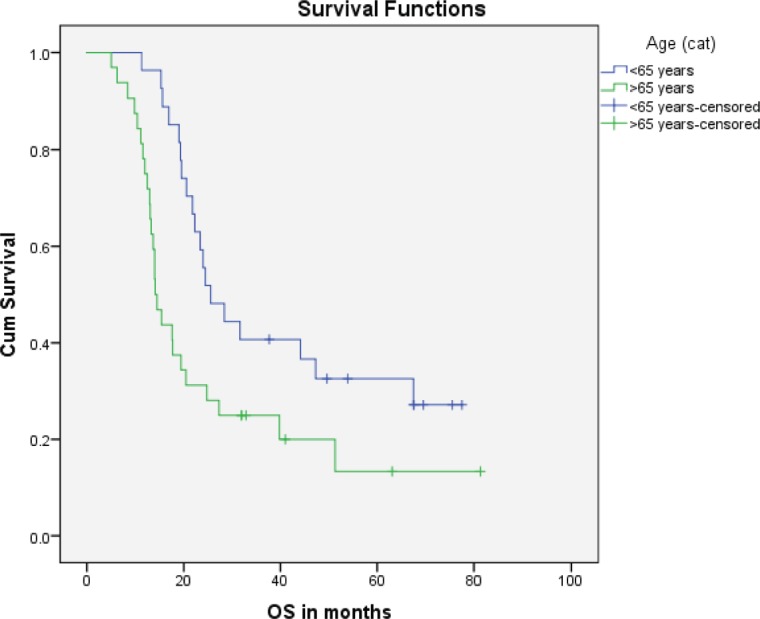
Kaplan–Meier curve for overall survival (in months) for patients <65 years versus ≥65 years.

**Figure 3.**
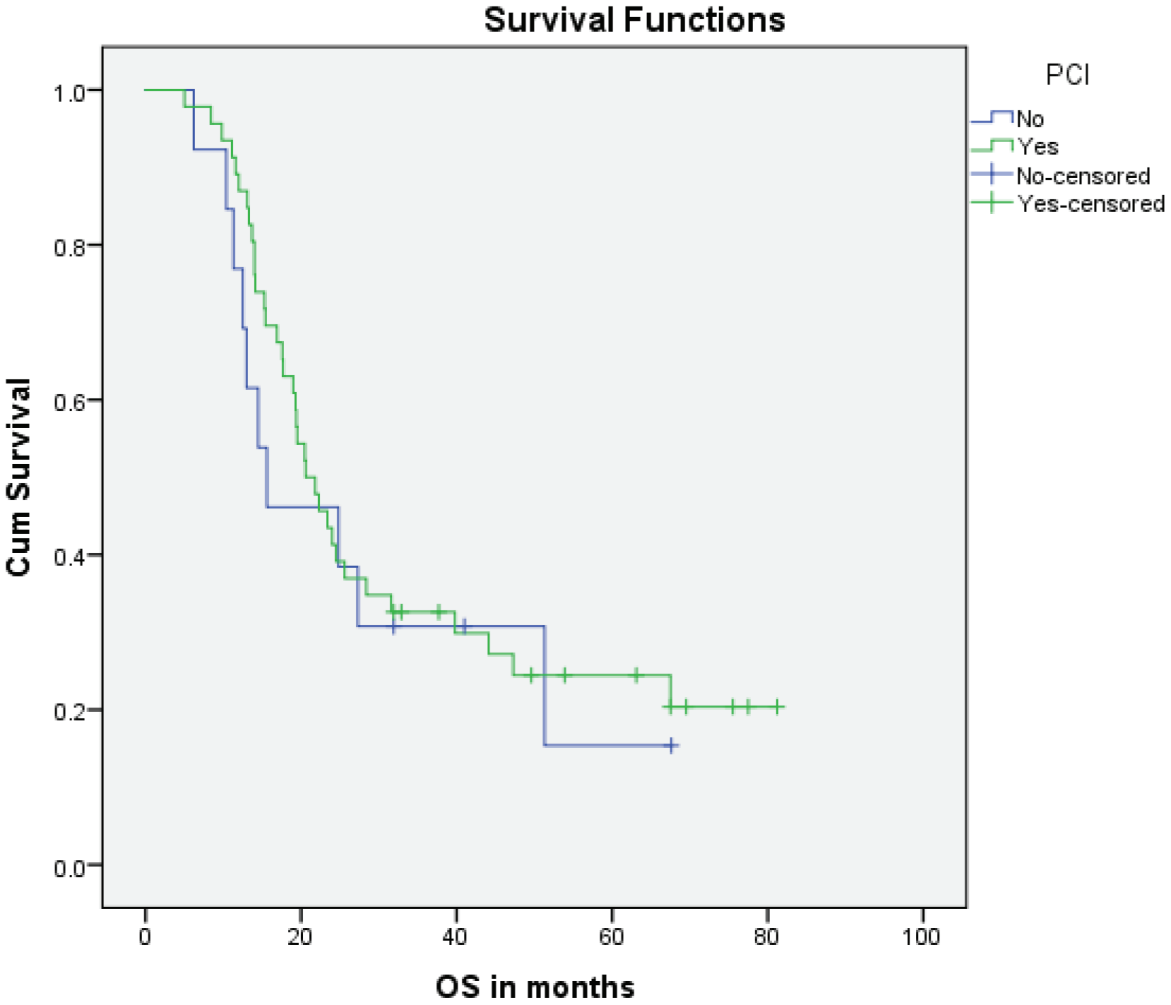
Kaplan–Meier curve for overall survival (in months) for patients who received PCI versus patients who didn’t.

Median PFS was 20.3 months (95% CI: 0–42.48, SE: 11.31) and was not significantly affected by gender (*p* = 0.268), age (*p* = 0.363), PS (*p* = 0.441), stage of disease (*p* = 0.571) or receipt of PCI (*p* = 0.680) (Figure 1). The median overall survival OS was 20.6 months (95% CI: 16.2–25.0) with a 1-year OS of 85%, 2-year OS was 42% and 3-year OS of 32%. Similarly, gender (*p* = 0.945), PS (*p* = 0.746), disease stage (*p* = 0.550) and PCI versus no PCI (*p* = 0.623) (Figure 3) didn’t impact on OS. The only significant factor for survival was age. For patients ≤65 years (*n* = 27), median OS was 25.6 months (95%CI: 18.1–33.1) versus 14.1 months (95% CI: 12.2–16.0) for patients (*n* = 32) >65 years (*p* = 0.013, Hazard Ratio 0.473, (95% CI: 0.26–0.85)) (Table 2, Figure 2).

## Literature search

### Sequential chemoradiotherapy (seqCRT) compared to concurrent chemoradiotherapy (cCRT)

Two studies focused on comparing the difference in patients with LS-SCLC treated with either cCRT or sequential chemotherapy followed by TRT. In a phase III trial published in 2002 by Takada *et al* [5], the authors reported outcomes in patients with LS-SCLC treated with either cCRT or seqCRT. The study featured a large sample size of 231 patients who were randomised into the two groups. It showed improved survival (median OS of 27.2 months versus 19.7 months) with cCRT (*p* = 0.097). In another randomised control trial published in 1997 by Gregor *et al* [6], 335 patients were randomised between arms (sequential and concurrent) of the study. It was found that those receiving cCRT had a median survival of 14 months compared to 15 months for patients receiving seqCRT.

### SeqCRT compared to chemotherapy alone

Six studies were identified that investigated the impact of the addition of seqCRT versus chemotherapy alone. In 1988, Creech *et al* [7] reported results of a randomised control trial examining the benefit of sequential chemotherapy followed by TRT, 50 Gy in 25 fractions compared to chemotherapy alone. There was a significant survival advantage of having TRT (median OS 18.65 months versus 15.48 months; *p* = 0.003).

A retrospective study conducted by Lester *et al* [8] yielded similar results, with improved survival in patients who received thoracic radiotherapy over chemotherapy alone. The median survival for those receiving chemotherapy alone was 9.0 months compared to 17.7 months for those receiving seqCRT (*p* < 0.001). Rosenthal *et al* [9] also examined the impact of adding TRT to the survival of patients with LS-SCLC. It was found that the median OS with chemotherapy alone was 14 months compared to 18 months with seqCRT (*p* = 0.073). Additionally, another study by Souhami *et al* [10] found a marginal improvement of a median survival (14.2 months with seqCRT versus 13.0 months receiving chemotherapy only) but this difference was non-significant. Similar findings were demonstrated by Kies *et al* [11] with a non-significant improvement in OS for those receiving seqCRT compared to the control group receiving chemotherapy alone. However, 38 of 42 patients who did not receive TRT had local relapse as compared to 20 of the 36 patients who did receive TRT.

Contrary to the above findings, one study showed no benefit of adding thoracic radiotherapy. Lebeau *et al* [12] reported that the median survival for chemotherapy alone was 496 days compared to a median survival of 316 days for sequential radiotherapy, however, this result was not statistically significant (*p* = 0.66). Similarly, in a small randomised study by Carlson *et al* (1991) [13], 24 patients received seqCRT. There was no significant difference in survival between those patients who received or didn’t receive TRT. However, addition of TRT did reduce the incidence of local recurrences (29% versus 58%; *p* = 0.042).

Two meta-analysis published in 1992 analysed the role of addition of TRT in LS-SCLC. Warde *et al* [14] showed a modest 2-year OS benefit (20% with TRT as compared to 15% without TRT; the odds ratio was 1.53 with 95% CI: 1.30–1.76, *p* < .001). A second meta-analysis published by Pignon *et al* [15] showed that the addition of TRT reduced the risk of mortality by 14% (Hazard ratio 0.86 with 95% CI: 0.78–0.94, *p* = 0.001). Three-year OS benefit was 5.4 ± 1.4%.

### SeqCRT—optimal radiation dose fractionation

Various radiation dose fractionations, ranging from 32 to 50 Gy were used in the studies. From the studies included, 10 provided data on the median survival. It is clear from the literature that multiple dose fractionation regimens of sequential TRT exist within current practice. From the ten studies included eight different regimes were used and have been listed in Table 3 (in comparison to the current study).

### Radiotherapy related toxicities

#### Pulmonary toxicity

Three studies highlighted pulmonary side effects amongst their patients receiving sequential TRT. Souhami *et al* [10] found that 35% of these patients experienced radiation induced pulmonary fibrosis with 40% of these patients having clinically evident dyspnoea. Additionally Ohnoshi *et al* [16] found that two patients died of radiation pneumonitis in the sequential arm of their trial. Similar results were obtained by Lebeau *et al* [12] who found that three patients had pneumonitis with one patient being clinical dyspnoeic as a result (4%).

#### Oesophageal toxicity

Three studies reported patients experiencing oesophagitis. Takada *et al* [5] found that 4% of patient receiving seqCRT experienced severe oesophagitis (compared to 9% in cCRT arm). This was also found in Lebeau [12] with ten cases of severe oesophagitis reported in the seqCRT arm of the study and also by Souhami *et al* [10] who found that oesophagitis was a common side effect amongst those receiving sequential radiotherapy.

## Discussion

A systematic review by Fried *et al* [17] included both cCRT and seqCRT found a significantly increased survival at 2 years for early TRT versus late TRT (HR 1.17 (95% CI: 1.02–1.35, *p* = 0.03). Since the publication of Turisi *et al* study [18], cCRT with twice daily radiotherapy has become the standard of care in LS-SCLC.

For patients who are deemed unfit for cCRT, addition of sequential TRT to chemotherapy is a feasible option with literature suggesting a modest survival benefit but significantly better local control with acceptable toxicity. The majority of the relevant studies are from the 20th century and used old radiotherapy techniques and the optimal TRT dose fractionation remains unknown. Modern imaging techniques have led to stage migration where some patients with extensive stage would previously have been classified as limited stage. Furthermore, with modern radiotherapy techniques it is possible to treat larger fields radically.

Our study demonstrated median survival of 20.3 months compared to 30 months (95% CI: 24–34) in twice daily concurrent chemoradiotherapy arm of a recently reported study (CONVERT) [19]. Similarly the two year survival was 42% in our series compared to 56% (95% CI: 50%–62%) in CONVERT study. Whilst inferior, seqCRT remains an option in patients who seemed unfit for cCRT and can result in reasonable survival in this patient population. In our series, age was associated with an adverse outcome using 65 years as cut off. This is in contrast to a second analysis of CONVERT study which showed no impact of age using cut of 70 years [20].

The treatment paradigm for extensive stage SCLC has changed with the use of atezolizumab in combination of carboplatin and etoposide in the first line setting [21]. TRT was not allowed in this study. Ongoing studies are assessing the potential impact of adding checkpoint inhibitors to concurrent chemoradiotherpay. Studies to investigate the potential benefit of checkpoint inhibitors in less fit patients receiving seqCRT should also be considered.

## Conclusion

The literature surrounding the use of sequential chemoradiotherapy is sparse and mostly published in the last century using old fashioned radiotherapy techniques with a range of dose and schedules. Our data showed inferior outcomes to the convert trial, which was performed in a similar time scale, as expected in this less fit patient population. However, it demonstrates reasonable outcomes and may be a valid treatment options in patients not suitable for concurrent treatment.

## Conflict of interest

None declared.

## Funding

No funding was received for this research study.

## Figures and Tables

**Table 1.** Reasons of not having concurrent chemoradiotherapy.

Reason	Number of patients	%
Co-morbidities	21	35.6
Poor performance status	15	25.4
Too large tumour bulk	10	16.9
Frailty	5	8.5
Suspicious of metastases on baseline imaging*	5	8.5
Not stated	2	3.4
Patient declined	1	1.7

* Lesions not felt to be malignant on imaging post chemotherapy.

**Table 2.** Prognostic factors affecting the overall survival demonstrated as median with 95% confidence intervals.

Characteristic n	Numbers 59	Results	p value
Male:female	30:29	Male: 20.5 months (95% CI 14.32–26.67) Female: 20.6 months (95% CI 12.33–28.86)	0.94
Age <65 versus ≥65 years	27 versus 32	Age ≤65: 25.6 months (95%CI 18.13–33.06) Age >65: 14.1 months (95% CI 12.21–15.98)	0.013
PS 0–1: 2–4	35 versus 24	PS 0 -1 19.3 months (95% CI 11.76–26.83) PS 2 – 4: 20.6 months (95% CI 15.19–26.00)	0.74
Stage I-IIIa versus IIIb	15 versus 44	Stage I-IIIa: 19.4 months (95%C 13.84–24.95) Stage IIIb: 20.6 month (95% CI 13.77–27.42)	0.55
PCI No vs Yes	13 versus 46	No PCI: 15.6 months (95%CI 1.74–29.45) Received PCI: 20.6 months (95% CI 16.16–25.03)	0.62

**Table 3.** Dose fractionation used in studies using thoracic radiotherapy in limited stage small cell lung cancer with median overall survival achieved.

Radiotherapy regimen	Studies used in	Median OS (months)
50 Gy in 25 fractions	Creech *et al* (1981-85) [7]	18.65
50 Gy in 20 fractions	Gregor *et al* (1989-95) [6]	15.00
48 Gy in 22 fractions	Kies *et al* (1980-83) [11]	18.50
45 Gy in 30 fractions	Takada *et al* (2002) [5]	20.00
40 Gy in 20 fractions	Ohnoshi *et al* (1981-86) [16] Souhami *et al* (1979-82) [10] Rosenthal *et al* (1977-79) [9]	15.74
40 Gy in15 fractions	Lester *et al* (2000-2002) [8]	17.70
32 Gy in 8 fractions	Lebeau *et al* (1986-88) [12]	11.29
55 Gy in 30 fractions	Carlson *et al* (1991) [13]	11.60
50 Gy in 25 fractions	Current study	20.60
